# Methplotlib: analysis of modified nucleotides from nanopore sequencing

**DOI:** 10.1093/bioinformatics/btaa093

**Published:** 2020-02-13

**Authors:** Wouter De Coster, Endre Bakken Stovner, Mojca Strazisar

**Affiliations:** b1 VIB, Center for Molecular Neurology, Antwerp 2610, Belgium; b2 Department of Computer Science, Norwegian University of Science and Technology, Trondheim 7013, Norway; b3 Department of Clinical and Molecular Medicine, Norwegian University of Science and Technology, Trondheim 7013, Norway

## Abstract

**Summary:**

Modified nucleotides play a crucial role in gene expression regulation. Here, we describe methplotlib, a tool developed for the visualization of modified nucleotides detected from Oxford Nanopore Technologies sequencing platforms, together with additional scripts for statistical analysis of allele-specific modification within-subjects and differential modification frequency across subjects.

**Availability and implementation:**

The methplotlib command-line tool is written in Python3, is compatible with Linux, Mac OS and the MS Windows 10 Subsystem for Linux and released under the MIT license. The source code can be found at https://github.com/wdecoster/methplotlib and can be installed from PyPI and bioconda. Our repository includes test data, and the tool is continuously tested at travis-ci.com.

**Supplementary information:**

Supplementary data are available at *Bioinformatics* online.

## 1 Introduction

Epigenetic covalent nucleotide modifications, which do not alter the primary DNA sequence, have many functions including transposon repression, expression regulation during development, imprinted expression and X-chromosome silencing (Gigante *et al.*, 2019; Greenberg and Bourc’his, 2019), and are known to play a role in many cellular functions, development and pathological states such as psychiatric disorders and neurodegeneration (Armstrong *et al.*, 2019; Gaine *et al.*, 2019). Over 40 verified types of modifications have been described, of which 5-methylcytosine (5mC) and N6-methyladenine (m6A) are the most studied (Sood *et al.*, 2019). The long-read sequencing platforms from Oxford Nanopore Technologies (ONT) enable genome-wide direct observation of modified nucleotides by assessing deviating current signals, for which multiple tools have been developed (Liu et al., 2019a, b; McIntyre *et al.*, 2019; Rand *et al.*, 2017; Simpson *et al.*, 2017; Stoiber *et al.*, 2016), but a comprehensive evaluation of their performance is lacking. For a recent review, we refer the reader to Xu and Seki (2019). To the best of our knowledge, no flexible visualization method is tailored to this type of data.

## 2 Materials and methods

We developed methplotlib, a software package for the visualization of the modified frequency and the per-read per-nucleotide probability of the presence of a nucleotide modification, together with additional summary overviews. While most work has been done on methylation, visualization using our tool is essentially agnostic to the type of nucleotide modification used as input, and future work may train upstream tools to recognize, e.g., hydroxymethylation or various RNA modifications in direct RNA sequencing (Garalde *et al.*, 2018; Leger *et al.*, 2019). At the time of writing, no community-standard format for nucleotide modifications has been established. The current methplotlib version is compatible with tab-separated files from nanopolish (Simpson *et al.*, 2017) or nanocompore (Leger *et al.*, 2019), and modifications encoded with MM/MP tags according to the SAM specifications. The API can straightforwardly be expanded to accommodate data in other formats. Gene and transcript annotation is extracted from a GTF file, and other types of annotations can be added in BED format.

**Fig. 1.**
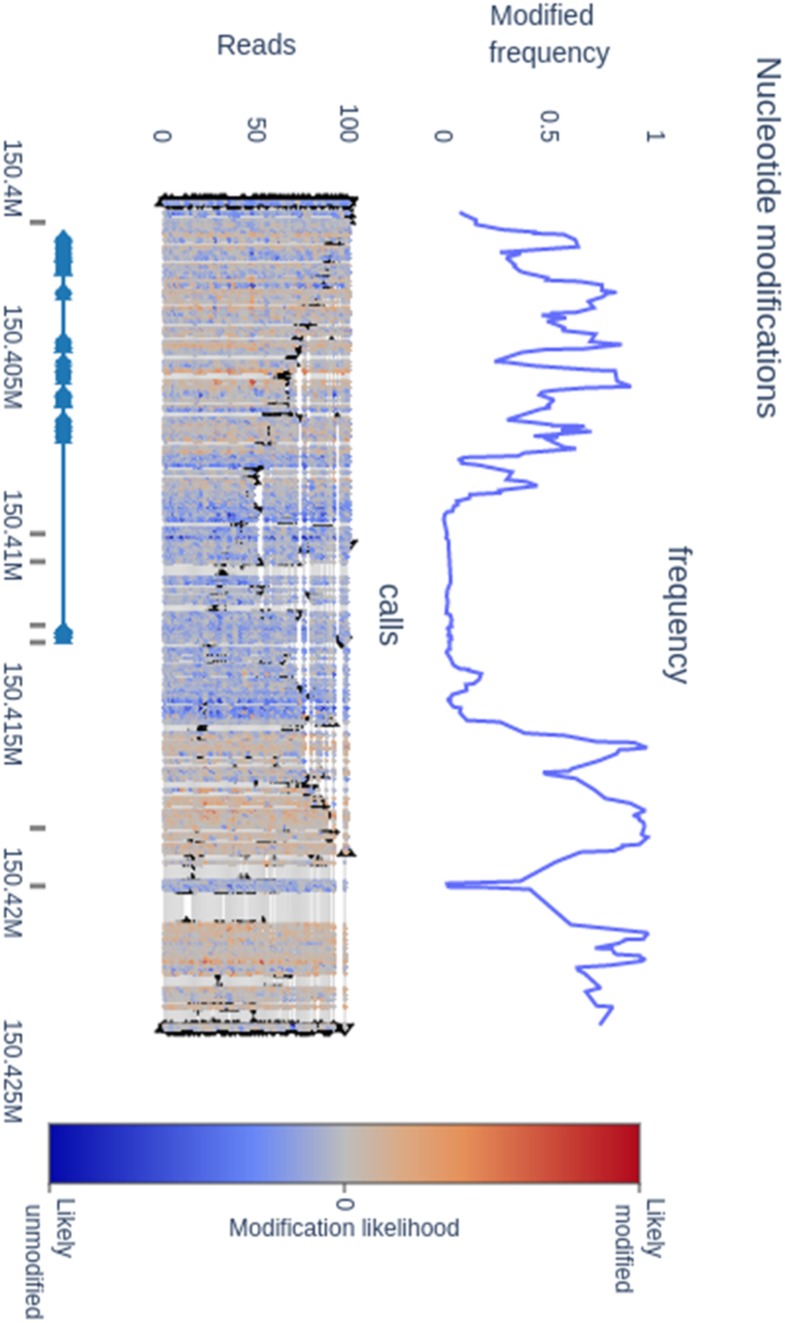
CpG methylation around the highly expressed CD74 gene. The top panel shows per position the frequency of methylated nucleotides, the middle panel shows per read per position the likelihood of a modified nucleotide with a color gradient and the bottom panel contains gene structure annotation and DNase hypersensitive regions. Regulatory regions show lower frequency of methylation

Our methplotlib tool depends on core Python modules and numpy (van der Walt *et al.*, 2011), pandas (McKinney, 2011), scikit-learn (Pedregosa *et al.*, 2011), pyranges (Stovner and Sætrom, 2019), pyfaidx (Shirley *et al.*, 2015) and plotly (Plotly Technologies Inc., 2015). We made our software easily available through PyPI and bioconda (Grüning *et al.*, 2018). Visualizations are created by default in dynamic HTML format or in other static output formats such as png, pdf and SVG, and show, optionally for multiple samples, (i) the raw likelihood of nucleotide modification per position per read, (ii) the frequency of having a modified nucleotide per position and (iii) an annotation track, showing the exon and gene structure. The examples (Fig. 1 and Supplementary Figs) were created using nanopolish call-methylation (Simpson *et al.*, 2017) of ONT PromethION data from a lymphoblastoid cell line of the Yoruban reference individual NA19240 (De Coster *et al.*, 2019) using gene annotation from Ensembl (Frankish *et al.*, 2019) and DNase hypersensitivity from ENCODE (ENCODE Project Consortium, 2012). Generation of the example plot (23 kbase locus) in Figure 1 takes <30 s and generates a 1.2 Mb dynamic HTML plot or a 146 Kb static PNG plot. A 10× larger region takes 50 s and results in an 11 Mb HTML file. As such, per read probabilities are best suited for gene-level visualization. Limiting the output to the frequency of modification without per read information is notably faster, leads to smaller files and as such is suitable for larger regions. While other genome browsers such as IGV (Thorvaldsdóttir *et al.*, 2013) and GenomeBrowse provide similar functionality to some extent for e.g. plotting the frequency of modified positions, the visualization of the per read probability is a feature unique to methplotlib, and furthermore, our implementation works out of the box for multiple file formats, such as recently introduced tags in the SAM format.

In addition, quality control plots are produced, including a principal component analysis to identify outliers, a pairwise correlation plot, highlighting more similar samples (Supplementary Fig. S2), box plots of global modification frequencies and a bar chart of all positions for which modifications were identified. Together with the tool, we have also developed a snakemake workflow (Koster and Rahmann, 2012) to facilitate the processing of multiple datasets and multiple regions of interest. A companion script annotate_calls_by_phase.py is included to separate the modification results in both paternal haplotypes using a phased bam file from WhatsHap haplotag (Martin *et al.*, 2016). Using phased modification calls allows us to detect allele-specific modification, statistically implemented using a Fisher exact test aggregating over a regulatory region (e.g. DNase hypersensitivity mark) in allele_specific_modification.py. This identifies mainly promoters affected by X-chromosome silencing (Supplementary Fig. S3) and multiple known imprinted genes including GNAS/GNAS-AS (Supplementary Fig. S4; Weinstein *et al.*, 2010), HYMAI1/PLAGL1 (Iglesias-Platas *et al.*, 2013) and HERC3/NAP1L5 (Cowley *et al.*, 2012). In larger cohorts, this approach could be used for the identification of methylation quantitative trait loci. The same approach is straightforwardly expanded to differential modification testing in differential_modification.py, for example to test epigenetic differences between patients and unaffected subjects.

## 3 Conclusion

Long-read sequencing technologies of ONT and PacBio enable for the first-time genome-wide direct observation of multiple types of nucleotide modifications without chemical modifications or affinity purification. To facilitate research in this emerging field we have developed methplotlib, a tool for the visualization of per read raw nucleotide modification probabilities or aggregated frequencies derived from nanopore sequencing. Our package additionally includes a scalable workflow, quality control plots and scripts for statistical analysis. The API supports nanopolish, nanocompore and CRAM format, and can straightforwardly be expanded to use emerging data formats and multiple types of nucleotide modifications as identified by upstream software.

## Supplementary Material

btaa093_Supplementary_Data
