# Supplementary material for: Methplotlib: analysis of modified nucleotides from nanopore sequencing
Source: Bioinformatics. 2020 Feb 13;36(10):3236–8. doi: 10.1093/bioinformatics/btaa093 (PMC7214038; doi:10.1093/bioinformatics/btaa093)
Supplement: btaa093_Supplementary_Data [file btaa093_supplementary_data.pdf]

Supplementary figures

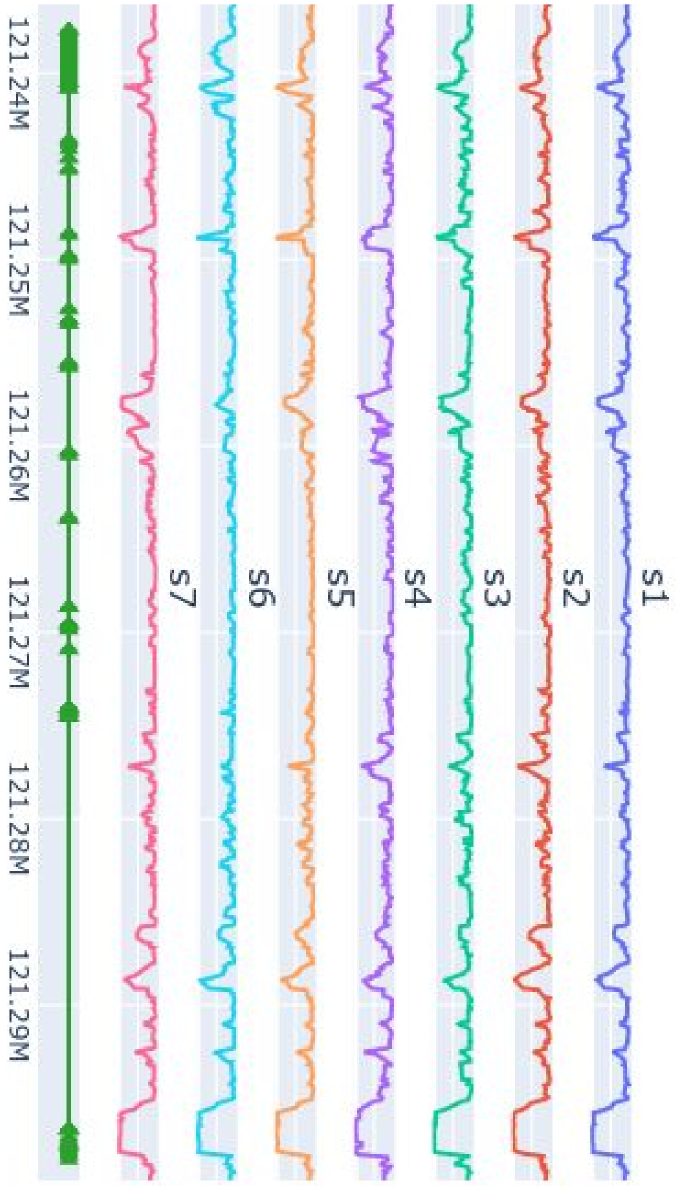

Supplementary Figure S1: methylated frequency of multiple samples
This plot shows the methylated frequency of 7 samples across the CAMKK2 gene.

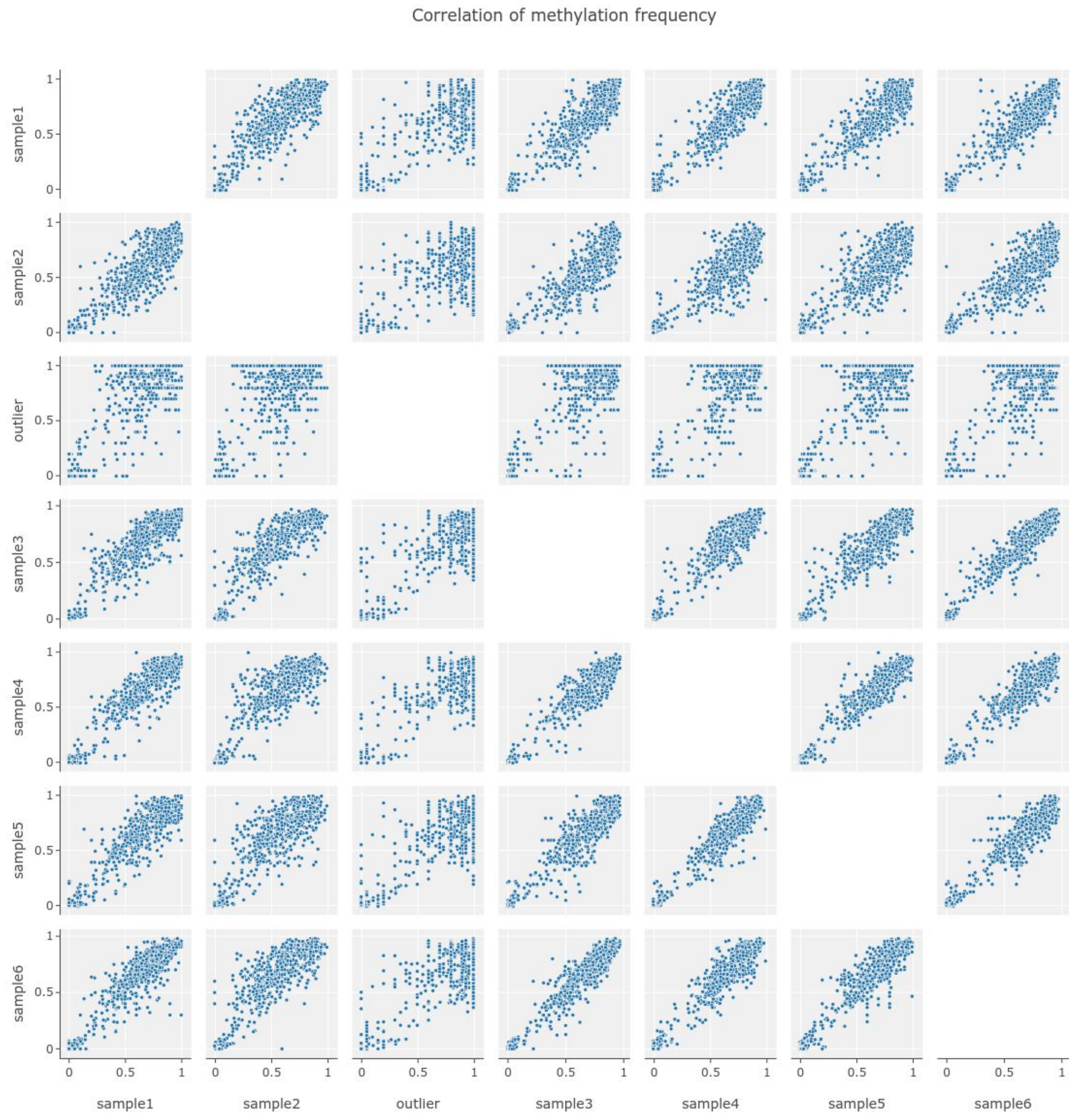

Supplementary Figure S2: pairwise correlation plot
Correlation of modification frequency across multiple datasets is plotted for each pair in the dataset, showing similarity for most samples with one notable outlier from a different tissue. All samples were sequenced on ONT PromethION.

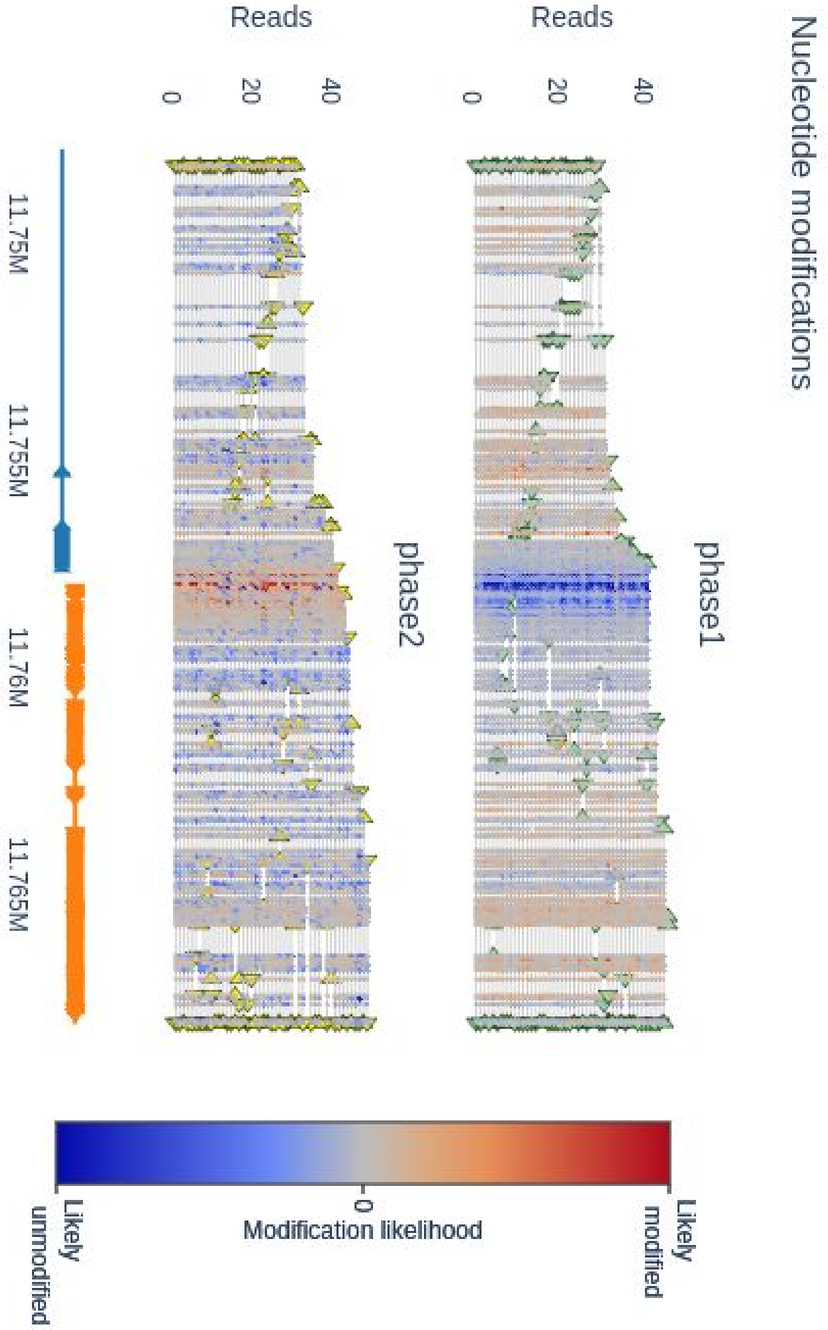

Supplementary Figure S3: X-chromosome silencing
Example of the MSL3 and AC004554.2 promoter, reads phased with WhatsHap

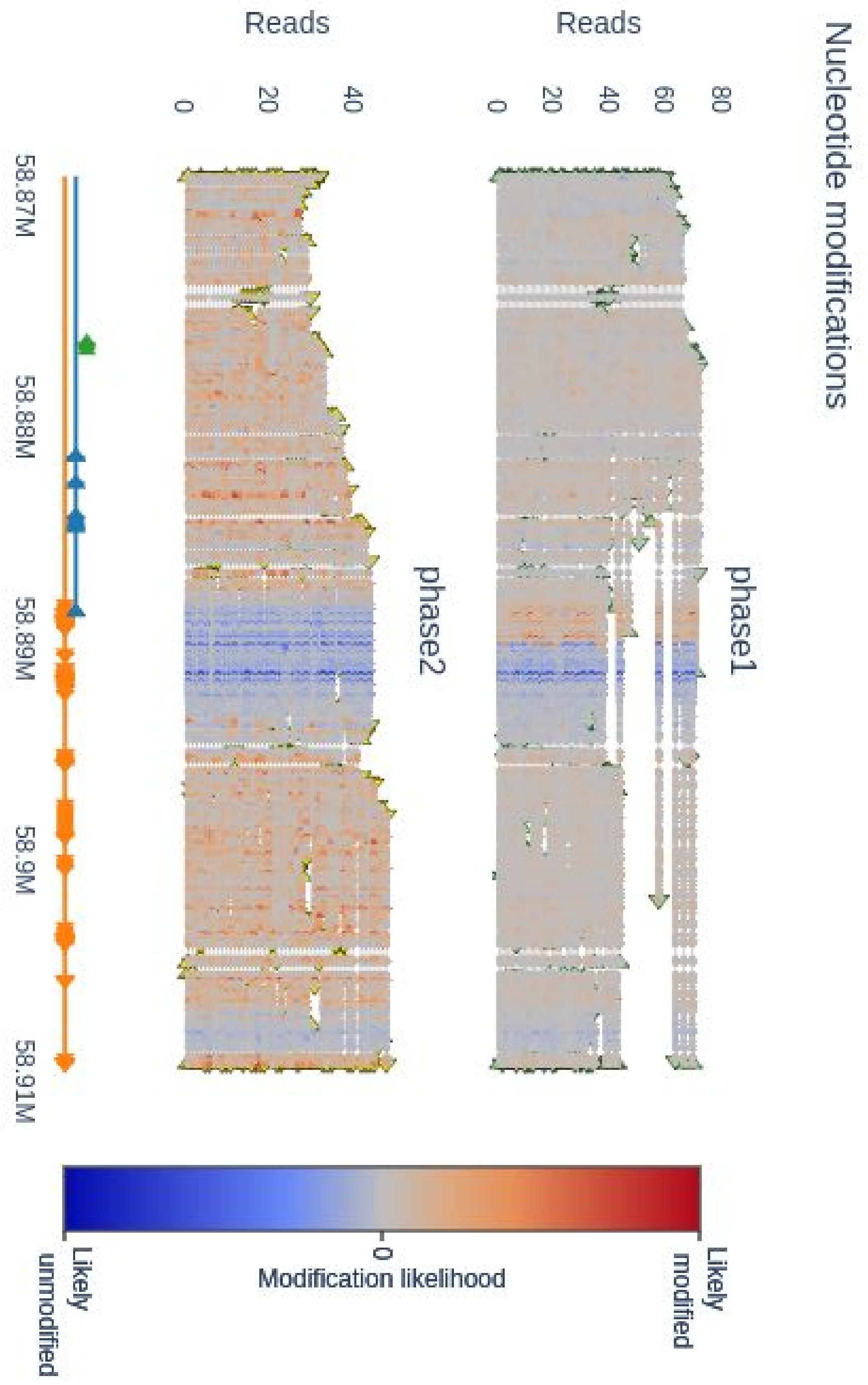

Supplementary Figure S4: genomic imprinting
Example of the GNAS/GNAS-AS locus, reads phased with WhatsHap
